# Time-cumulated blood pressure exposure and incident impairment of glucose tolerance and diabetes mellitus

**DOI:** 10.1186/s12872-017-0537-y

**Published:** 2017-05-02

**Authors:** Yun Tao Wu, Lu Song, Xiao Xue Liu, Jing Sheng Gao, Xiao Ming Zheng, Chun Yu Ruan, Hai Yan Zhao, Shuo Hua Chen, Wen Yuan Gao, Jost B. Jonas, Shou Ling Wu

**Affiliations:** 1 School of Pharmaceutical Science and Technology, Tianjin University, Tianjin, China; 2 Department of Cardiology, Kailuan Hospital, North China University of Science and Technology, Tangshan, 063000 China; 3 Graduate school, North China University of Science and Technology, Tangshan, China; 4 Department of Cardiology, Tangshan People’s Hospital, North China University of Science and Technology, Tangshan, China; 5 Department of Health Care Center, Kailuan Hospital, North China University of Science and Technology, Tangshan, China; 6 Department of Ophthalmology, Medical Faculty Mannheim of the Ruprecht-Karls-University of Heidelberg, Heidelberg, Germany

**Keywords:** Incident glucose tolerance impairment, Incident diabetes, Cumulative blood pressure exposure, Kailuan study

## Abstract

**Background:**

With the marked increase in the prevalence of diabetes mellitus, it was the purpose of our study to assess a potential association of time-cumulated exposure to systolic (CumSBP) and of diastolic blood pressure (CumDBP) with onset of impaired glucose tolerance and diabetes mellitus.

**Methods:**

The prospective investigation included participants of the longitudinal Kailuan Study with three baseline examinations in 2006–2007, 2008–2009 and 2010–2011, re-examination in 2012–2013, and no diabetes mellitus at baseline. Cumulative blood pressure (BP) was calculated as cumBP = [(BP_1_ + BP_2_)/2 × time_1–2_] + [(BP_2_ + BP_3_)/2 × time_2–3_]. Based on cumSBP, the study population was stratified into four groups (cumSBP < 480mmHgxyear;*n* = 15,339; 480mmHgxyear ≤ cumSBP < 520mmHgxyear;*n* = 7214; 520mmHgxyears ≤ cumSBP < 560mmHgxyears;*n* = 5675; and cumSBP ≥ 560mmHgxyears;*n* = 10,576).

**Results:**

After adjusting for demographic, anthropomorphic, biochemical, socioeconomic and lifestyle parameters and as compared with the first group, the second, third and fourth group showed a significantly higher incidence of diabetes (*P*-trend < 0.001;hazard ratio (HR);95% confidence interval (CI):1.28(1.08,1.51),1.54(1.29,1.84), and 2.33(1.98,2.73), respectively), higher incidence of impairment of glucose tolerance (*P*-trend < 0.001;HR;95% CI1.17(1.02,1.33), 1.43(1.25,1.64), and 2.09(1.85,2.37), respectively), and higher incidence of diabetes developing out of an impairment of glucose tolerance (*P*-trend < 0.001;HR;95% CI:1.22(0.97,1.54),1.47(1.16,1.86), and 2.01(1.62,2.50), respectively). An increase in cumSBP by 10 mmHg/year or an increase in cumDBP by 5 mmHg/year was associated with a hazard ratio of incident diabetes of 1.04 (95% CI:1.03,1.04) and 1.02(1.02,1.03), respectively, with a hazard ratio of incident impairment of glucose tolerance of 1.04(95% CI:1.03,1.04) and 1.03(95% CI:1.02,1.03), respectively, and with a hazard ratio of incident diabetes developing from impairment of glucose tolerance of 1.04(95% CI:1.03,1.04) and 1.03(95% CI:1.02,1.03), respectively.

**Conclusions:**

Time-cumulated exposure to elevated blood pressure was significantly associated with an elevated incidence of impaired glucose tolerance and diabetes.

**Electronic supplementary material:**

The online version of this article (doi:10.1186/s12872-017-0537-y) contains supplementary material, which is available to authorized users.

## Background

According to the recent Global Burden of Disease Study 2015, diabetes mellitus has become one of the most common causes for disability and premature death [1, 2]. Major sequelae of diabetes include an increased risk of arterial hypertension among other disorders such as cardiovascular diseases and cerebrovascular events [3, 4]. Previous studies suggested that hypertension may vice versa increase the risk of developing diabetes mellitus, while other studies contradicted such a relationship [5–9]. One of the reasons for the discrepancy between the studies may have been that most of the studies did not assess blood pressure in a longitudinal manner so that the cumulative exposure to blood pressure could not be taken into account. Cumulative exposure to a risk factor as compared to a time point-related measurement of the risk factor may however be a more precise parameter if the potential associations of the risk factor are assessed. To cite examples, the prospective study by the U.K. Prospective Diabetes Study Group was one of the first to suggest that the cumulative exposure to elevated glucose serum concentrations increased the risk of diabetes-related complications [10]. Navar-Boggan and colleagues showed that the cumulative exposure to hyperlipidemia increased the incidence of coronary heart diseases [11]. A study by Zemaintis and associates revealed that a high cumulative blood pressure exposure was related with an increased risk of renal complications [12]. Since only few studies have so far been focused on the association between cumulative blood pressure exposure and incident diabetes or incident glucose tolerance impairment, we conducted this study and analyzed the relationship between cumulative blood pressure exposure and both diabetes-related parameters in the Kailuan Study.

## Methods

The community of Kailuan in Tangshan in the Chinese province of Hebei was the site for the Kailuan study which has been a prospective cohort study registered under No. #: ChiCTR-TNC-11001489. An informed consent was signed by all study participants and the Ethics Committees of Kailuan General Hospital approved the investigation. The study included employees and retirees of a coal mine company (Kailuan Group Company). At baseline, the study population consisted of 101,510 individuals with an age ranging between 18 years and 98 years. The study participants were repeatedly and prospectively examined in two-years intervals [13–15].

Trained interviewers asked the study participants questions focusing on the socioeconomic background, physical activity, diet, and known systemic diseases. Blood pressure, body height, body weight and hip and waist circumference were measured and electrocardiography and an ultrasound examination of the pancreas, spleen, kidney, liver, gallbladder, abdomen and breast were carried out. The serum concentration of glucose, high-density lipoproteins, low-density lipoproteins and triglycerides was determined in blood samples taken under fasting conditions. We measured the blood pressure between 7 am and 9 am at the same day when all other examinations were performed. Before the blood pressure measurements were taken, the individuals had to sit in a relaxed manner for at least 15 min and had to have refrained from taking tea or coffee and from smoking for at least half an hour. Using a mercury sphygmomanometer, blood pressure was measured at the right upper arm, with the measurement being repeated twice in intervals of 2 min. We took the mean of all three measurements for further statistical analysis.

The cumulative exposure to blood pressure was calculated by multiplying the exposure doses with exposure time, i.e., cumulative blood pressure (cumBP) = [(BP_1_ + BP_2_) / 2 x time_1–2_] + [(BP_2_ + BP_3_) /2 x time_2–3_], where BP1, BP2 and BP3 were the blood pressure measured at the first, second and third physical examination, respectively; time_1–2_ and time_2–3_ meant the interval between the two blood pressure determinations [11]. cumBP was differentiated between the cumulative systolic blood pressure (cumSBP) and the cumulative diastolic blood pressure (cumDBP).

The definition of current smoking consisted of inhalation of at least one cigarette daily during the preceding year, and consumption of alcohol was defined as the daily intake of at least 50 mL of alcohol daily during the last year. Individuals were considered to carry out physical exercise if they weekly performed three or more exercises of at least half an hour. A fasting serum glucose concentration of ≥7.0 mmol/L, history of diabetes or current therapy of diabetes with intake of blood glucose-lowering drugs were the criteria for the diagnosis of diabetes mellitus. A fasting serum concentration of glucose of ≥6.1 mmol/L and <7.0 mmol/L without history of diabetes and without blood-glucose lowering therapy formed the definition of an impairment of the glucose tolerance.

Inclusion criteria for the current investigation were participation in the baseline examinations in the years 2006–2007, 2008–2009 and 2010–2011, and in the follow-up examination in 2012–2013; an age of ≥18 years at the first baseline examination; and available blood pressure measurements and measurements of the diabetes-related parameters data from all four examinations. Exclusion criterion was the diagnosis of diabetes (i.e., history of diabetes or currently taking hypoglycemic drugs or a fasting serum glucose concentration of ≥7.0 at any of the three baseline examinations)**.**

For the statistical analysis we used the first three examinations performed in 2006–2007, 2008–2009 and 2010–2011 as baseline examinations, and the follow-up period was the time of the third baseline examination and the fourth examination in the period from 2010 to 2011. Endpoint events were the development of an impairment of glucose tolerance without previously detected impairment of glucose tolerance, the development of diabetes without previously detected diabetes, and the incidence of diabetes developing from an impairment of glucose tolerance. The measurements were presented as mean ± standard deviations. First, we carried out a univariate analysis of the associations of incident impairment of glucose tolerance or of incident diabetes with other parameters. The cumulative incidence of the endpoint events, i.e. development of an impairment of glucose tolerance or development of diabetes, in dependence of the cumSBP and cumDBP was calculated using the life-cycle method. The difference in the incidence of the endpoint events was compared with the Log-rank test. Hazard ratios (HR) and their 95% confidence intervals (CI) were calculated by the Cox proportion hazard model and natural spline function. A result was considered to be statistically significant if *P*-value was less than 0.05 (two-sided test).

## Results

**Fig. 1:**
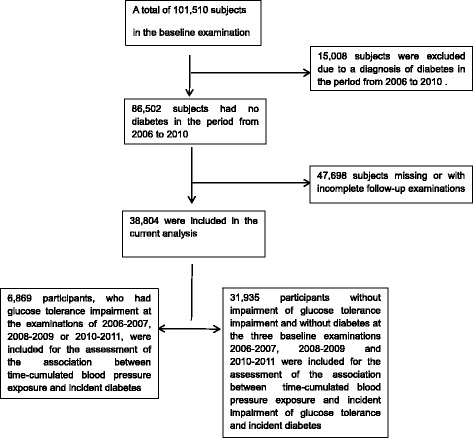
Scheme showing the inclusion and exclusion of study participants

Out of 101,510 individuals participating in the baseline examination of the Kailuan Study, 15,008 subjects were excluded since they already had a diagnosis of diabetes prior to the examination in the period from 2012 to 2013. Due to missing or incomplete follow-up examinations, we additionally excluded 47,698 individuals, so that eventually a total of 38,804 participants were included into the current analysis. For the examination of the relationship between cumBP and glucose tolerance impairment, we included 31,935 participants who did not show glucose tolerance impairment in 2006, 2008 or 2010. For the assessment of the association between cumBP and risk of converting from glucose tolerance impairment to diabetes, we included the remaining 6869 participants who showed glucose tolerance impairment at the examinations in the periods of 2006 to 2007, 2008 to 2009 or 2010 to 2011 (Fig. 1).

The individuals included into the current analyses as compared to the individuals excluded due to missing or incomplete follow-up examinations were significantly (*P* ≤ 0.001) younger, had a higher level of education, a higher concentration of high-density lipoproteins, a lower resting heart rate, systolic blood pressure and diastolic blood pressure, lower concentration of fasting blood glucose, total cholesterol, low-density lipoproteins, high-sensitive C-reactive protein and uric acid, and a lower prevalence of male gender, performing of physical exercise and intake of pressure-lowering drugs (Additional file 1: Table S1).

The mean age of the study participants (*n* = 38,804 (29,129 (75.1%) men)) was 47.8 ± 11.7 years (Additional file 1: Table S1). The whole study population was stratified into four groups based on the cumSBP. The first group included 15,339 individuals with a cumSBP of <480 mmHg x year (120 mmHg × 4 years), the second group consisted of 7214 subjects with a cumSBP ≥480 mmHg x year and <520 mmHgxyear (or 130 mmHg × 4 years), the third group included 5675 participants with a cumSBP ≥520 mmHg x years and <560 mmHg x years (or 140 mmHg × 4 years), and the fourth group consisted of 10,576 individuals with a cumSBP ≥560 mmHg x years (Table 1). Compared with the first group, the second, third and fourth group showed a significantly (*P* ≤ 0.001) higher percentage of male gender, older age, higher heart rate, higher body mass index, higher serum concentration of glucose, total cholesterol, high-density lipoproteins, low-density lipoproteins, uric acid, triglycerides, and high-sensitive C-reactive protein, higher prevalence of smoking and alcohol consumption, higher prevalence of physical exercise, and higher prevalence of blood pressure lowering drugs, and lower level of education (Table 1).

**Table 1:** Characteristics (Mean ± Standard deviation) of the study participants stratified by the cumulative systolic blood pressure

Variables	Total population (*n* = 38,804)	Group 1 (*n* = 15,339)	Group 2 (*n* = 7214)	Group 3 (*n* = 5675)	Group 4 (*n* = 10,576)	*P*-value
Cumulative Systolic Blood Pressure (mmHg x year)		<480	≥480 and <520	≥520 and <560	≥560	
Men (n)	29,129 (75.1%)	10,461 (68.2%)	5813 (80.6%)	4528 (79.8%)	8327 (78.7%)	<0.001
Age (Years)	47.82 ± 11.70	41.96 ± 9.75	46.54 ± 10.38	50.16 ± 10.64	55.94 ± 10.51	<0.001
Heart Rate (Beats/min)	73.03 ± 9.69	72.46 ± 9.16	73.26 ± 9.64	73.26 ± 9.86	73.58 ± 10.32	<0.001
Systolic Blood Pressure (mmHg)	126.9 ± 19.3	115.1 ± 12.7	125.4 ± 13.7	130.9 ± 15.9	143.1 ± 19.8	<0.001
Diastolic Blood Pressure (mmHg)	82.1 ± 11.2	76.4 ± 8.8	82.1 ± 9.4	84.4 ± 10.3	88.9 ± 11.7	<0.001
Body Mass Index (kg/m^2^)	24.89 ± 3.42	24.08 ± 3.31	25.06 ± 3.34	25.35 ± 3.39	25.71 ± 3.39	<0.001
Fasting Serum Concentration of Glucose (mmol/L)	5.01 ± 0.65	4.96 ± 0.62	5.05 ± 0.65	5.04 ± 0.66	5.02 ± 0.68	<0.001
Total Cholesterol (mmol/L)	4.89 ± 1.11	4.75 ± 1.05	4.87 ± 1.15	4.96 ± 1.14	5.07 ± 1.14	<0.001
High-Density Lipoproteins (mmol/L)	1.55 ± 0.39	1.54 ± 0.37	1.54 ± 0.38	1.57 ± 0.42	1.57 ± 0.42	<0.001
Low-Density Lipoproteins (mmol/L)	2.29 ± 0.89	2.26 ± 0.82	2.33 ± 0.86	2.30 ± 0.91	2.30 ± 1.01	<0.001
Uric Acid Concentration (μmol/L)	284.7 ± 82.1	270.6 ± 77.3	284.0 ± 79.6	288.5 ± 81.3	303.3 ± 86.8	<0.001
Triglycerides (Median (Q1,Q3))	1.23 (0.86, 1.84)	1.12 (0.78, 1.63)	1.26 (0.89, 1.89)	1.31 (0.93, 1.98)	1.35 (0.96, 2.02)	<0.001
High-Sensitive C-Reactive Protein (Median (Q1,Q3))	0.70 (0.27, 1.96)	0.60 (0.20, 1.50)	0.69 (0.25, 1.90)	0.70 (0.30, 2.02)	0.96 (0.38, 2.60)	<0.001
Smoking (n)	11,616 (29.9%)	4423 (28.8%)	2359 (32.7%)	1772 (31.2%)	3062 (29.0%)	<0.001
Drinking (n)	6608 (17.0%)	1986 (12.9%)	1323 (18.3%)	1076 (19.0%)	2223 (21.0%)	<0.001
Exercise (n)	5212 (13.4%)	1244 (8.1%)	779 (10.8%)	831 (14.6%)	2358 (22.3%)	<0.001
Pressure-lowering drugs (n)	3211 (8.3%)	226 (1.5%)	292 (4.0%)	464 (8.2%)	2229 (21.1%)	<0.001
Education: Illiteracy / Primary (n)	2655 (6.8%)	473 (3.1%)	404 (5.6%)	426 (7.5%)	1352 (12.8%)	<0.001
Junior High School (n)	26,706 (68.8%)	10,294 (67.1%)	5093 (70.6%)	4083 (71.9%)	7236 (68.4%)	
High School (n)	6264 (16.1%)	2864 (18.7%)	1158 (16.1%)	818 (14.4%)	1424 (13.5%)	
College or Higher (n)	3179 (8.2%)	1708 (11.1%)	559 (7.7%)	348 (6.1%)	564 (5.3%)	

**Fig. 2:**
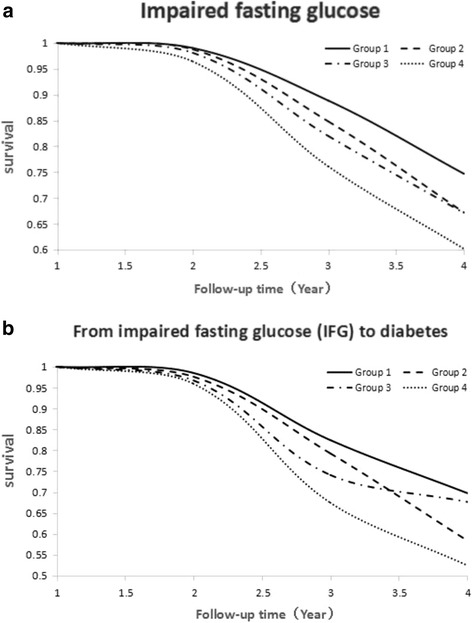
**a** Development of Impairment of Glucose Tolerance in 2563 (8.0%) Individuals out of 31,935 Participants Without Impairment of Glucose Tolerance and Without Diabetes at the Baseline Examinations. **b** Development of New-Onset of Diabetes in 874 (12.7%) Individuals out of 6869 Participants with Impaired Glucose Tolerance at the Baseline Examinations

Within a follow-up period of 2.22 ± 0 38 years after the third baseline examination of 2010–2011, 1523 (3.9%) individuals out of the 38,804 participants without diabetes (with or without glucose tolerance impairment) at the baseline examinations newly developed diabetes mellitus; 2563 (8.0%) individuals out of the 31,935 participants without impairment of glucose tolerance and without diabetes at the baseline examinations newly developed an impaired glucose tolerance; and 874 (12.7%) individuals out of 6869 participants with impaired glucose tolerance newly developed diabetes mellitus. The risk of an incident impairment of glucose tolerance and of incident diabetes significantly increased with increasing cumulative blood pressure exposure as indicated by an increasing group number of the stratified study population (Additional file 2: Table S2; Tables 2 and 3) (Fig. 2).

**Table 2:** Multiple COX regression model for the time-cumulated exposure to systolic blood pressure and to diastolic blood pressure in relation to the new-onset of impairment of glucose tolerance in 2563 (8.0%) individuals out of 31,935 participants without impairment of glucose tolerance and without diabetes at the baseline examinations

New-onset of impairment of glucose tolerance in participants without impairment of glucose tolerance and without diabetes at the baseline	Quartiles of cumulative systolic blood pressure (cumSBP)				Each increase in cumSBP by 10 mmHg·/ year	*P*-trend
	Q1	Q2	Q3	Q4		
cumSBP (Median) (mmHg x Year)	439.1	498.6	538.4	614.1		
Number of Participants (n)	775 (5.8%)	439 (7.5%)	399 (8.8%)	950 (11.8%)		<0.001
Model 1	1.00	1.30 (1.15, 1.50)	1.60 (1.41, 1.82)	2.42 (2.17, 1.82)	1.04 (1.03, 1.04)	<0.001
Model 2	1.00	1.29 (1.14, 1.45)	1.56 (1.37, 1.78)	2.32 (2.07, 2.60)	1.03 (1.03, 1.04)	<0.001
Model 3	1.00	1.17 (1.02, 1.33)	1.43 (1.25, 1.64)	2.09 (1.85, 2.37)	1.03 (1.03, 1.04)	<0.001
New-onset of impairment of glucose tolerance in participants without impairment of glucose tolerance and without diabetes at the baseline	Quartiles of cumulative diastolic blood pressure (cumDBP)				Each increase in cumDBP by 5 mmHg·/ year	*P*-trend
	Q1	Q2	Q3	Q4		
cumDBP (Median) (mmHg x Year)	239.1	329.6	349.4	388.4		
Number of Participants (n)	875 (6.1%)	391 (7.6%)	388 (9.0%)	909 (11.1%)		<0.001
Model 1	1.00	1.26 (1.11, 1.42)	1.55 (1.37, 1.75)	2.35 (2.12, 2.60)	1.03 (1.03, 1.03)	<0.001
Model 2	1.00	1.24 (1.10, 1.40)	1.53 (1.35, 1.73)	2.27 (2.05, 2.52)	1.03 (1.02, 1.03)	<0.001
Model 3	1.00	1.12 (0.98, 1.27)	1.35 (1.18, 1.54)	2.02 (1.80, 2.25)	1.02 (1.02, 1.03)	<0.001

CumSBP: Q 1:cumSBP < 480mmHgxyear,Q 2:480mmHgxyear ≤ cumSBP < 520mmHgxyear,Q 3:520mmHgxyear ≤ cumSBP < 560mmHgxyear,Q 4: cumSBP ≥ 560mmHgxyear

CumDBP: Q 1:cumDBP < 320mmHgxyear,Q 2:320mmHgxyear ≤ cumDBP < 340mmHgxyear,Q 3:340mmHgxyear ≤ cumDBP < 360mmHgxyear,Q 4: cumDBP ≥ 360mmHgxyear

Model 1: adjusted for sex and age (years)

Model 2: adjusted for model 1 and further adjusted for smoking, drinking, exercises, education and taking pressure-lowering drugs

Model 3: adjusted for model 2 and further adjusted for heart rate, body mass index, and fasting serum concentration of glucose, triglycerides, high-sensitive C-reactive protein, high-density lipoproteins, low-density lipoproteins and uric acid concentration

**Table 3:** Multiple COX regression model for the time-cumulated exposure to systolic blood pressure and to diastolic blood pressure in relation to the new-onset of diabetes in 874 (12.7%) individuals out of 6869 participants with impaired glucose tolerance at the baseline examinations

New-onset of diabetes in participants with impaired glucose tolerance at the baseline examinations	Quartiles of cumulative systolic blood pressure (cumSBP)				Each increase in cumSBP by 10 mmHg·/ year	*P*-trend
	Q1	Q2	Q3	Q4		
cumSBP (Median) (mmHg x Year)	446.9	498.9	539.2	620.4		
Number of Participants (n)	181 (9.7%)	148 (11.1%)	155 (13.4%)	390 (15.5%)		<0.001
Model 1	1.00	1.32 (1.06, 1.64)	1.69 (1.35, 2.10)	2.45 (2.00, 2.99)	1.04 (1.03, 1.04)	<0.001
Model 2	1.00	1.34 (1.08, 168)	1.70 (1.36, 2.12)	2.37 (1.93, 2.92)	1.04 (1.03, 1.04)	<0.001
Model 3	1.00	1.22 (0.97, 1.54)	1.47 (1.16, 1.86)	2.01 (1.62, 2.50)	1.03 (1.03, 1.04)	<0.001
New-onset of diabetes in participants with impaired glucose tolerance at the baseline examinations						
	Q1	Q2	Q3	Q4	Each increase in cumDBP by 5 mmHg·/ year	*P*-trend
cumDBP (Median) (mmHg x Year)	299.2	329.5	349.5	391.8		
Number of Participants (n)	228 (10.5%)	123 (11.2%)	139 (12.4%)	384 (15.5%)		<0.001
Model 1	1.00	1.17 (0.93, 1.46)	1.45 (1.17, 1.80)	2.40 (2.01, 2.85)	1.03 (1.03, 1.04)	<0.001
Model 2	1.00	1.19 (00.95, 1.49)	1.48 (1.19, 1.84)	2.36 (1.97, 2.82)	1.03 (1.03, 1.04)	<0.001
Model 3	1.00	1.10 (0.87, 1.39)	1.28 (1.02, 1.60)	2.06 (1.70, 2.49)	1.03 (1.02, 1.03)	<0.001

CumSBP: Q 1:cumSBP < 480mmHgxyear,Q 2:480mmHgxyear ≤ cumSBP < 520mmHgxyear,Q 3:520mmHgxyear ≤ cumSBP < 560mmHgxyear,Q 4: cumSBP ≥ 560mmHgxyear

CumDBP: Q 1:cumDBP < 320mmHgxyear,Q 2:320mmHgxyear ≤ cumDBP < 340mmHgxyear,Q 3:340mmHgxyear ≤ cumDBP < 360mmHgxyear,Q 4: cumDBP ≥ 360mmHgxyear

Model 1: adjusted for sex and age (years)

Model 2: adjusted for model 1 and further adjusted for smoking, drinking, exercises, education and taking pressure-lowering drugs

Model 3: adjusted for model 2 and further adjusted for heart rate, body mass index, and fasting serum concentration of glucose, triglycerides, high-sensitive C-reactive protein, high-density lipoproteins, low-density lipoproteins and uric acid concentration

We then applied a Cox proportion risk model with the incidence of an impaired glucose tolerance or incidence of diabetes as dependent variable and with cumSBP and cumDBP as independent variables. In a model 1, we adjusted for gender and age; in model we additionally adjusted for smoking, alcohol consumption, amount of physical exercises, level education and taking of blood pressure-lowering drugs; and in model, we additionally adjusted for heart rate, body mass index, and fasting serum concentrations of glucose, triglycerides, high-sensitive C-reactive protein, high-density lipoproteins, low-density lipoproteins and uric acid. The Cox proportion risk model revealed that an increase in cumSBP by 10 mmHg/year or an increase in cumDBP by 5 mmHg/year was associated with a hazard ratio of incident diabetes of 1.04 (95% CI: 1.03, 1.04) and 1.02 (95% CI: 1.02, 1.03), respectively. The same increases in cumSBP and in cumDBP were associated with a hazard ratio of incident impairment of glucose tolerance of 1.04 (95% CI: 1.03, 1.04) and 1.03 (95% CI: 1.02, 1.03), respectively. The same increases in cumSBP and in cumDBP were associated with a hazard ratio of incident diabetes developing from an impairment of glucose tolerance of 1.04 (95% CI: 1.03, 1.04) and 1.03 (95% CI: 1.02, 1.03), respectively (Additional file 2: Table S2; Tables 2 and 3).

## Discussion

The prospective Kailuan Study showed that the exposure to elevated blood pressure accumulated over 3 examinations performed in 5 years was significantly associated with an increased incidence of diabetes, within the subgroup of individuals without previously impaired glucose tolerance and within the subgroup of individuals with previously impaired glucose tolerance. In a parallel manner, a higher accumulated exposure to elevated blood pressure was correlated with a higher rate of newly developed impairment of glucose tolerance (Additional file 2: Table S2; Tables 1, 2 and 3). For each increase in the cumSBP by 10 mmHg/year the risk of incident diabetes and of incident impairment of glucose tolerance increased by 3%. In multivariate analysis, this increase was independent of other diabetes-related related risk factors such as older age, gender, smoking, alcohol consumption, amount of physical exercise, level education and taking blood pressure-lowering drugs, and heart rate, body mass index, and fasting serum concentrations of glucose, triglycerides, high-sensitive C-reactive protein, high-density lipoproteins and low-density lipoproteins and uric acid.

The findings obtained in our study were in agreement with the results found in previous investigations. In a study Emdin and colleagues on more than 4 million adults, an increase in systolic blood pressure by 20 mmHg was correlated with a 58% higher risk of developing diabetes, and an increase in diastolic blood pressure by 10 mmHg was correlated with a 52% higher chance to develop diabetes [7]. The association between blood pressure and the incidence of diabetes mellitus (type 2) was also examined by Hayashi and colleagues [5] In a prospective study on more than 7000 Japanese men with an age ranging between 35 years and 60 years, high normal blood pressure as compared with normal blood pressure was associated with a 39% higher risk, and arterial hypertension was associated with a 76% higher risk, to develop diabetes mellitus. In the Candesartan Antihypertensive Survival Evaluation in the Japan trial on high-risk Japanese hypertensive patients, Yasuno and associates found that for each standard deviation increase in pulse pressure the hazard ratio for the development of diabetes was 1.44 in a multiple regression analysis [6]. A longitudinal investigation performed by Stahl and coworkers included more than 7000 men who were free of diabetes and who were followed-up for 35 years [16]. The hazard ratio for the incidence of diabetes was 1.43, 1.43 and 1.95 for individuals with a systolic blood pressure of 130–139 mmHg, 140–159 mmHg, and ≥160 mmHg, respectively, while a systolic blood pressure of less than 130 mmHg served as reference level. Analyzing the data of the Atherosclerosis Risk in Communities study, the Coronary Artery Risk Development in Young Adults study and the Framingham Heart Study offspring cohort together, Wei and colleagues found that the age-adjusted incidence of type 2 diabetes was increasingly higher across increasing blood pressure groups [17]. In contrast to the studies mentioned above, blood pressure factors was not predictive for incident diabetes in a study by Norberg and associates, whose investigation was a case-referent study nested within a population-based health survey [8]. In a similar manner in the investigation by Wang and colleagues, systolic and diastolic blood pressure did not predict the onset of diabetes [9].

Our study adds to the current knowledge on the association between hypertension and the development of diabetes and an impairment of glucose tolerance by taking into account the time-cumulative exposure to an increased blood pressure over a relatively long time of 5 years as baseline values, and by also addressing the development of an impairment of glucose tolerance. Studies on the associations between the time-cumulative exposure to risk factors for the incidence of major diseases have so far been performed with respect to the cumulative data on hyperglycemia and diabetic complications, the cumulative exposure to high serum concentrations of cholesterol and the incidence of coronary heart disease, and to the cumulative blood pressure exposure and the development of kidney damage [10–12].

The reasons for the association between time-cumulated blood pressure exposure and incident impairment of glucose tolerance and incident diabetes mellitus have remained elusive so far. Previous studies have shown that a low-grade inflammatory process occurs in both diabetes and hypertension both of which could be considered as chronic inflammatory diseases [18–21]. Correspondingly, inflammatory markers such as C-reactive protein are increased in patients with diabetes and in patients with arterial hypertension and do also predict the development of these diseases [22, 23]. Gene regulatory network analysis has demonstrated that oxidative stress is important for diabetes and hypertension, with the oxidative stress-mediated regulation cascade being the common mechanistic link among the pathogenesis of diabetes, arterial hypertension, and other related inflammatory diseases [24]. Also, insulin as a pleiotropic hormone plays a pivotal role in the development of arterial hypertension and diabetes.

In view of the pronounced increase in the prevalence of diabetes and arterial hypertension, the search of new screening tools that are economic and easily available in the clinical practice is of high practical importance. To date, several markers have been available to indicate an increased risk for the onset and progression of diabetes, such as inflammatory markers or proinsulin/insulin ratio, however they are not easily feasible for large scale screening [25, 26]. Furthermore, early identification of diabetes may be useful in terms of lifestyle intervention and prevention of long term complications. The results of the present study suggest that the time-cumulated exposure to elevated blood pressure is an additional risk factor predicting an increased likelihood to develop an impaired glucose tolerance and diabetes. Time-cumulated exposure to elevated blood pressure in combination with other parameters such as inflammatory markers, the proinsulin/insulin ratio, body mass index and amount of physical activity may be taken in a synopsis to estimate the risk of eventual onset of diabetes.

Limitations of our study should be discussed. First, the follow-up period of 2.22 years after the baseline period of 5 years was relatively short. With a longer follow-up and a higher number of patients developing diabetes or an impairment of glucose tolerance, the statistical significance of the associations between hypertension and the incidence might have become even clearer. Second, it has remained elusive, whether the multivariate analysis with adjustment for a multitude of parameters (including gender, age, smoking, alcohol consumption, amount of physical exercises, level education, taking of blood pressure-lowering drugs, heart rate, body mass index and fasting serum concentrations of glucose, triglycerides, high-sensitive C-reactive protein, high-density lipoproteins and low-density lipoproteins and uric acid) had not overlooked an additional factor potentially confounding the associations between hypertension and incidence of diabetes and impairment of glucose tolerance. In view of the robust statistical significance of these associations however, it might have been unlikely that a factor besides those already taken into account might have markedly impeded the associations between hypertension and incidence of diabetes and impairment of glucose tolerance as found in our study.

## Conclusions

Time-cumulated exposure to elevated blood pressure was significantly associated with an elevated incidence of impaired glucose tolerance and diabetes, after adjusting for serum concentration of glucose and other diabetes-related parameters. Diabetes mellitus may be included into the list of sequelae of arterial hypertension. Future studies may address whether therapeutic lowering of elevated blood pressure may prevent or delay the onset of impaired glucose tolerance and diabetes.

## Additional files

Additional file 1: Table S1. Characteristics of Individuals Included into the Study as Compared to the Individuals Excluded from the Study Due to Missing or Incomplete Follow-Up Examinations. (DOCX 30 kb)

Additional file 2: Table S2. Multiple COX Regression Model for the Time-Cumulated Exposure to Systolic Blood Pressure and to Diastolic Blood Pressure in Relation to the New-Onset of Diabetes in 1523 (3.9%) Individuals out of 38,804 Participants Without Diabetes (With or Without Glucose Tolerance Impairment) at the Baseline Examinations. (DOCX 30 kb)

## Abbreviations

BP: Blood pressure
CI: Confidence interval
CumDBP: Time-cumulated exposure to diastolic blood pressure
CumSBP: Time-cumulated exposure to systolic blood pressure
HR: Hazard ratio
