# Supplementary material for: Time-cumulated blood pressure exposure and incident impairment of glucose tolerance and diabetes mellitus
Source: BMC Cardiovasc Disord. 2017 May 2;17:106. doi: 10.1186/s12872-017-0537-y (PMC5414153; doi:10.1186/s12872-017-0537-y)
Supplement: Supplementary file 1 — Characteristics of Individuals Included into the Study as Compared to the Individuals Excluded from the Study Due to Missing or Incomplete Follow-Up Examinations. (DOCX 30 kb) [file 12872_2017_537_MOESM1_ESM.docx]

Additional file 1: Table S1. Characteristics of Individuals Included into the Study as Compared to the Individuals Excluded from the Study Due to Missing or Incomplete Follow-Up Examinations

| Variables | Included subjects (n=38,804) | Excluded subjects (n=47,698) | Total population  (n=86,502) | *P*-Value |
| --- | --- | --- | --- | --- |
| Men (n) | 29,129 (75.1%) | 39,499 (82.8%) | 68,628 (79.3%) | <0.001 |
| Age (Years) | 47.82 ± 11.70 | 54.13 ± 13.06 | 51.30 ± 12.86 | <0.001 |
| Heart Rate (Beats/min) | 73.03 ± 9.69 | 73.60 ± 10.14 | 73.34 ± 9.94 | <0.001 |
| Systolic Blood Pressure (mmHg) | 126.9 ± 19.3 | 132.1 ± 21.6 | 131.1 ± 21.1 | <0.001 |
| Diastolic Blood Pressure (mmHg) | 82.1 ± 11.2 | 83.7 ± 12.0 | 83.5 ± 11.8 | <0.001 |
| Body Mass Index (kg/m^2^) | 24.89 ± 3.42 | 24.78 ± 3.47 | 24.83 ± 3.45 | <0.001 |
| Fasting Serum Concentration of Glucose (mmol/L) | 5.01 ± 0.65 | 5.06 ± 0.69 | 5.04 ± 0.67 | <0.001 |
| Total Cholesterol (mmol/L) | 4.89 ± 1.11 | 4.93 ± 1.13 | 4.91 ± 1.12 | <0.001 |
| High-Density Lipoproteins (mmol/L) | 1.55 ± 0.39 | 1.54 ± 0.41 | 1.55 ± 0.40 | 0.002 |
| Low-Density Lipoproteins (mmol/L) | 2.29 ± 0.89 | 2.37 ± 0.90 | 2.33 ± 0.90 | <0.001 |
| Uric Acid Concentration (µmol/L) | 28465 ± 82.1 | 295.0 ± 84.3 | 290.3 ± 83.5 | <0.001 |
| Triglycerides (Median  (Q1,Q3) | 1.23 (0.86, 1.84) | 1.22 (0.87, 1.82) | 1.22 (0.87, 1.83) | 0.54 |
| High-Sensitive C-Reactive Protein, (Median (Q1,Q3)) | 0.70 (0.27, 1.96) | 0.81 (0.30, 2.16) | 0.78 (0.30, 2.08) | <0.001 |
| Smoking (n) | 11,616 (29.9%) | 14,301 (30.0%) | 25,917 (30.0%) | 0.88 |
| Drinking (n) | 6,608 (17.0%) | 8,334 (17.5%) | 14,942 (17.3%) | 0.09 |
| Exercise (n) | 5,212 (13.4%) | 7,413 (15.5%) | 12,625 (14.6%) | <0.001 |
| Intake of Pressure-Lowering Drugs (n) | 3,211 (8.3%) | 5,124 (10.7%) | 8,335 (9.6%) | <0.001 |
| Education | | | | |
| Illiteracy / Primary (n) | 2,655 (6.8%) | 6,412 (13.7%) | 9,067 (10.6%) | <0.001 |
| Junior High School (n) | 26,706 (68.8%) | 31,711 (67.8%) | 58,417 (68.3%) |  |
| High School (n) | 6,264 (16.1%) | 5,167 (11.1%) | 11,431 (13.4%) |  |
| College or Higher (n) | 3,179 (8.2%) | 3,470 (7.4%) | 6,649 (7.8%) |  |
