# Supplementary material for: Time-cumulated blood pressure exposure and incident impairment of glucose tolerance and diabetes mellitus
Source: BMC Cardiovasc Disord. 2017 May 2;17:106. doi: 10.1186/s12872-017-0537-y (PMC5414153; doi:10.1186/s12872-017-0537-y)
Supplement: Supplementary file 2 — Multiple COX Regression Model for the Time-Cumulated Exposure to Systolic Blood Pressure and to Diastolic Blood Pressure in Relation to the New-Onset of Diabetes in 1523 (3.9%) Individuals out of 38,804 Participants Without Diabetes (With or Without Glucose Tolerance Impairment) at the Baseline Examinations. (DOCX 30 kb) [file 12872_2017_537_MOESM2_ESM.docx]

Additional file 2: Table S2. Multiple COX Regression Model for the Time-Cumulated Exposure to Systolic Blood Pressure and to Diastolic Blood Pressure in Relation to the New-Onset of Diabetes in 1,523 (3.9%) Individuals out of 38,804 Participants Without Diabetes (With or Without Glucose Tolerance Impairment) at the Baseline Examinations

| New-Onset Diabetes in Participants Without Diabetes (With or Without Glucose Tolerance Impairment) at the Baseline Examinations | Quartiles of Cumulative Systolic Blood Pressure (cumSBP) | | | | Each Increase in cumSBP by 10 mmHg·/ Year | *P*-Trend |
| --- | --- | --- | --- | --- | --- | --- |
|  | Q1 | Q2 | Q3 | Q4 |  |  |
| cumSBP (Median) (mmHg x Year) | 439.3 | 498.7 | 538.4 | 615.8 |  |  |
| Number of Participants (n) | 392(2.6%) | 260 (3.6%) | 253 (4.5%) | 618 (5.8%) |  | <0.001 |
| Model 1 | 1.00 | 1.50 (1.28, 1.77) | 2.02 (1.72, 2.39) | 3.28 (2.83, 3.79) | 1.04 (1.04, 1.05) | <0.001 |
| Model 2 | 1.00 | 1.49 (1.27, 1.75) | 1.96 (1.66, 2.31) | 3.04 (2.62, 3.54) | 1.04 (1.04, 1.05) | <0.001 |
| Model 3 | 1.00 | 1.28 (1.08, 1.51) | 1.54 (1.29, 1.84) | 2.33 (1.98, 2.73) | 1.04 (1.03, 1.04) | <0.001 |
| New-Onset Diabetes in Participants Without Diabetes (With or Without Glucose Tolerance Impairment) at the Baseline Examinations | Quartiles of Cumulative Diastolic Blood Pressure (cumDBP) | | | | Each Increase in cumDBP by 5 mmHg·/ Year | *P*-Trend |
|  | Q1 | Q2 | Q3 | Q4 |  |  |
| cumDBP (Median) (mmHg x Year) | 294.0 | 329.6 | 349.5 | 389.1 |  |  |
| Number of Participants (n) | 462 (2.8%) | 238 (3.8%) | 233 (4.3%) | 590 (5.5%) |  | <0.001 |
| Model 1 | 1.00 | 1.43 (1.22, 1.68) | 1.72 (1.47, 2.03) | 2.85 (2.49, 3.25) | 1.03 (1.03, 1.04) | <0.001 |
| Model 2 | 1.00 | 1.41 (1.20, 1.65) | 1.68 (1.43, 1.98) | 2.66 (2.32, 3.04) | 1.03 (1.03, 1.04) | <0.001 |
| Model 3 | 1.00 | 1.19 (1.00, 1.40) | 1.32 (1.11, 1.57) | 2.08 (1.80, 2.40) | 1.03 (1.02, 1.03) | <0.001 |

CumSBP: Q 1:cumSBP<480mmHgxyear,Q 2:480mmHgxyear≤cumSBP<520mmHgxyear,Q 3:520mmHgxyear≤cumSBP<560mmHgxyear,Q 4: cumSBP≥560mmHgxyear.

CumDBP: Q 1:cumDBP<320mmHgxyear,Q 2:320mmHgxyear≤cumDBP<340mmHgxyear,Q 3:340mmHgxyear≤cumDBP<360mmHgxyear,Q 4: cumDBP≥360mmHgxyear.

Model 1: adjusted for sex and age (years).

Model 2: adjusted for model 1 and further adjusted for smoking, alcohol consumption, exercises, level education and taking blood pressure-lowering drugs.

Model 3: adjusted for model 2 and further adjusted for heart rate, body mass index, and fasting serum concentrations of glucose, triglycerides, high-sensitive C-reactive protein, high-density lipoproteins and low-density lipoproteins and uric acid.
